# Differences in hip bone mineral density may explain the hip fracture pattern in osteoarthritic hips

**DOI:** 10.3109/17453670903039528

**Published:** 2009-06-01

**Authors:** Olof Wolf, Håkan Ström, Jan Milbrink, Sune Larsson, Hans Mallmin

**Affiliations:** Department of Orthopedics, Uppsala University HospitalSweden

## Abstract

**Introduction** In patients with osteoarthritis of the hip (OAH), trochanteric fractures are much more common than femoral neck fractures. One reason may be altered bone composition in the proximal femurs. OAH often leads to a fixed external rotation of the hip, leading to difficulties in positioning during DXA measurements. We compared BMD in OAH-affected legs and healthy legs.

**Patients and methods** 40 patients with strictly unilateral OAH were cross-sectionally investigated with DXA at the hips and heels bilaterally as well as body composition of the legs. 3 regions of interest in the proximal femur were measured: femoral neck (FN), trochanter (TR), and total hip (TH). The design of the study allowed us to perform paired t-test between the OAH side and the healthy side.

**Results** BMD was increased by 4.1% in FN, and reduced by 8.3% in TR and 4.1% in TH (p < 0.001 for all comparisons).

**Interpretation** The differences in BMD, with decrease in the trochanter and increase in the femoral neck, may offer an explanation for the pattern of hip fractures seen in osteoarthritis. External rotation of the hip cannot explain the differences in BMD.

## Introduction

Reduced weight bearing due to pain from osteoarthritis of the hip (OAH) could lead to disuse of the affected lower limb and result in lower bone mineral density (BMD) and muscle mass. Other studies have shown that OAH is associated with higher BMD only adjacent to the affected joint (Knight et al. 1992) or with generalized high BMD (Nevitt et al. 1995, Dequeker et al. 2003). If patients with osteoarthritic hips suffer from a hip fracture, it is commonly a trochanteric fracture (Stahl 1957, Weintroub et al. 1982, Middleton and Ferris 1996). The reason for this is unclear, although one explanation might be a reduced BMD at the trochanteric region but not at the femoral neck. The relative risk of hip fracture is increased 2.0–3.5 times for every standard deviation (SD) of reduction in proximal femoral BMD (Marshall et al. 1996, Johnell et al. 2005).

Positioning of the proximal femur is of importance for the accuracy of BMD measurements (Girard et al. 1994, Goh et al. 1995). Patients with osteoarthritic hips often have restricted motion, especially rotation and abduction/adduction, which could affect the results of such measurements.

We performed a cross-sectional study on patients with strictly unilateral OAH who were scheduled for total hip arthroplasty (THA). We conducted the study in order to evaluate the influence of OAH on BMD of the proximal femur, BMD at the heel, and body composition of the lower limb. Our hypothesis was that differences in BMD of the proximal femur in OAH could be one explanation for the higher proportion of trochanteric fractures in these patients. Our secondary hypothesis was that differences in body composition parameters between an OAH-affected limb and the healthy limb could relate to the degree of preoperative weight bearing, pain, or muscle strength in the hip.

## Material and methods

### Patients

The study was approved by the local ethics committee of Uppsala University (approval no. Ups 99242).

Patients with unilateral OAH who were 25–65 years old, with a body weight less than 110 kg, living in Uppsala municipality, and who were on the waiting list for a THA were eligible for the study. Exclusion criteria were cortisone medication or other medication known to affect bone metabolism, malignancy, previous hip surgery, or BMI above 35. 44 patients gave informed consent to participate and were included in the study between February 2000 and April 2003. The original study was designed to evaluate the stability of an uncemented total hip implant using radiostereometric analysis (Strom et al. 2007). The study protocol included preoperative X-rays and DXA measurements together with clinical evaluations, which allowed us to perform this cross-sectional study. In order to increase the strength of the diagnosis of unilateral OAH, we excluded 3 patients who underwent contralateral THA within 5 years of the primary procedure. In addition, 1 patient with avascular necrosis of the femoral head after internal fixation for a femoral neck fracture was excluded. Altogether, 40 patients (20 women) with a mean age of 55 (26–63) years and with bilateral measurements of lower limbs were eligible for this study.

With the amount of importance that is placed on standardized positioning of the leg during DXA scanning in order to maximize precision, the fixed external rotation commonly seen in arthritic hips might cause a systematic error. In order to address this problem, a second group of patients was recruited and their hips were scanned in different positions with respect to rotation. In all, 21 patients (16 women) with a mean age of 64 (41–83) years who were scheduled for routine bilateral proximal femur DXA measurement at Uppsala Osteoporosis Unit, Uppsala University Hospital, during October and November 2006 were included in this part of the project.

### Missing data and exclusions

Due to missing data on our 40 patients in the main study, 3, 2, and 2 patients were excluded from hip, heel, and body composition analysis, respectively. This left us with 37 patients with BMD of the proximal femur, 38 with BMD of the heels, and 38 patients with total body scan. In weight bearing, hip abductor strength, SF-36, Merle d'Aubigné, and pain report, we lacked data on 1 patient. There were complete sets of data on 35 patients.

### Main study

All patients were examined preoperatively with conventional radiography of the affected hip and the pelvis. The degree of radiographic osteoarthritis was classified according to the Kellgren/Lawrence global grading scale (Kellgren and Lawrence 1957). The patients were evaluated (1) by visual analog scale (VAS) for pain at rest and during weight bearing exercises, (2) by a Merle-d'Aubigné protocol (D'Aubigne and Postel 1954) in order to score pain, walking ability and range of motion, and (3) by a health-related quality of life rating instrument, the Swedish SF-36 (Sullivan et al. 1995). The height and weight were recorded. Hip abductor strength was measured with a dynamometer (CSD 400; Chatillon Inc., New York, NY). Measurements were repeated 5 times and the mean value in kg was used for statistical analysis. In order to evaluate the degree of weight bearing as a variable for severity of osteoarthritis, weight bearing was measured using shoes with sensor-equipped soles and analyzed with the F-scan system (Tekscan Inc., Boston, MA). Mean value in kg based on 3 recordings, each including 5 steps, was used. Comparisons were performed between the OAH-affected limb and the healthy limb, which served as a control.

Areal bone densitometry at the proximal femur, bilaterally, and total body composition measurements were performed with a pencil-beam total body DXA scanner: DPX-L (Lunar Co., Madison, WI). 3 regions of interest (ROIs) at the proximal femur were analyzed for areal bone mineral density (g/cm^2^): the femoral neck (FN), the trochanter region (TR), and the total hip (TH). In addition, we analyzed bone mineral content (BMC) and projected bone area in these regions. The proximal femur BMD ROIs were compared to age- and sex matched weight-adjusted US white reference populations (Z-score) provided by the manufacturer. The DXA images of patients were analyzed for obvious differences in hip rotation between the healthy hip and the OAH-affected hip. The long-term precision error for a lumbar spine reference phantom expressed as percentage coefficient of variance was less than 1% during the study period.

The heels were measured with cone-beam DXA equipment: PIXI (Lunar). A manufacturer-defined ROI of the heels was analyzed for areal BMD (g/cm^2^) of the calcaneus. Comparisons with sex-specific reference populations provided by the manufacturer were only available for T-scores from young adults.

Regional analyses of the total body measurements were performed for total lower limb mass (TLLM, g), fat mass (FM, g), lean tissue mass (LTM, g), and bone mineral content (BMC, g) of both lower limbs. Such absolute values are likely to be strongly influenced by the manually defined lower limb ROIs, since even small differences will affect the results. In order to reduce these influences, we also chose to compare body composition relationships as percentages of total limb mass, i.e. FM%, LTM%, and BMC%.

### Rotation group

In order to control for the effect of rotation on hip BMD, 21 patients without any hip joint disease (42 proximal femurs in total) were measured bilaterally at the proximal femur with a short fan beam DXA scanner (Prodigy; GE-Lunar) and analyzed for FN, TR, and TH. This was done as part of a first routine visit to the Uppsala Osteoporosis Unit, Uppsala University Hospital. The proximal femurs were scanned both at (1) the recommended standard position with the foot in approximately 10–15 degrees of internal rotation, i.e. zero rotation of the femoral neck, and (2) with the foot in a vertical position, i.e. with 10–15 degrees of external rotation of the femoral neck (Figure). The second position was chosen to simulate a common position—often a fixed external rotation—seen in patients with OAH. Comparisons between the two positions of rotations were made for BMD of the three proximal femur ROIs. The long-term precision error for a lumbar spine reference phantom (expressed as percentage coefficient of variance) was less than 1% during the study period.

**Figure 1.**
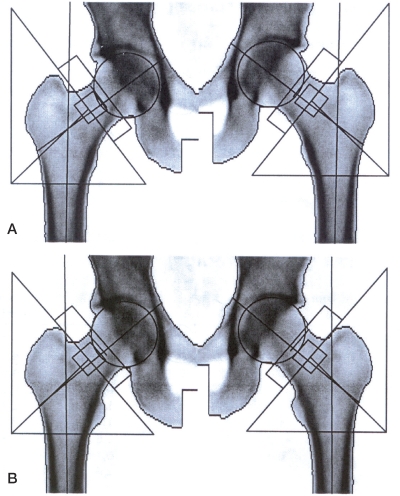
Zero rotation position (A) and 10–15° external rotation position (B) in bilateral femoral DXA scans. In the latter, the external rotation shows the prominence of the lesser trochanter.

### Statistics

*Main study.* The healthy leg served as control. For normally distributed data, we used paired t-test to compare mean values between the OAH side and the healthy side. Wilcoxon signed rank tests were performed for non-parametric data. Differences were considered significant when p < 0.05. Values are given as mean (SD). Statistical analysis was performed with Statistica software version 7.1 (StatSoft Inc., Tulsa, OK).

*Rotation group.* We used paired t-test to compare mean values of hip BMD between internal and external rotation. Differences were considered significant when p < 0.05. Values are given as mean (SD).

## Results

The patients in the main study, 20 men and 20 women, had an average age of 55 years and a mean BMI of 27. The Z-scores were above or close to zero, i.e. normal for gender and age, adjusted for weight. The patients in the rotation group, 5 men and 16 women, were 9 years older on average and had a mean BMI of less than 22 (Table 1).

**Table 1.** Characteristics of patients. Values are mean (SD)

	OAH	Rotation group
No. of patients	40	21
Age, years	55 (8.8)	64 (12)
Sex (M/F)	20/20	5/16
Weight, kg	80 (13)	72 (14)
Length, cm	172 (8.8)	167 (9.9)
BMI	27 (3.2)	22 (3.4)
Z-FN (n = 37)	0.77 (1.2)	0.05 (1.0)
Z-TR (n = 37)	–0.25 (1.2)	–0.4 (1.0)
Z-TH (n = 37)	0.12 (1.1)	–0.3 (0.9)
Z-Total Body	0.71 (1.1)	not measured

### Main study

All patients had OAH of grade 2 or higher, and most patients (> 90%) had grade 3 or 4 according to Kellgren and Lawrence. Patients with OAH had mean VAS scores for pain of 3.8 (2.2) and 6.6 (1.8) at rest and during weight bearing, respectively. The subscores for general health (GH), mental health (MH), and the mental component summary (MCS) corresponded with values from an age- and sex-matched Swedish reference population (Sullivan et al. 2002). Values for physical functioning (PF), role limitations due to physical function (RP), bodily pain (BP), vitality (VT), social functioning (SF), role limitations due to emotional problems (RE), and physical component summary (PCS) were markedly lower in the OAH group than in the reference population. The Merle-d'Aubigné scores were statistically significantly lower for the OAH-affected limbs.

*Weight bearing and muscle strength.* Preoperatively, equal amounts of weight bearing were registered for the OAH-affected limb and the control limb: 69 (13) kg and 70 (14) kg, respectively; nor was there any difference in hip abductor strength between sides: 22 (8) and 21 (8).

*Bone mineral density.* The proximal femur ROIs for the OAH-affected side and the control side were normal, with Z-scores of 0.77 (1.2) to –0.4 (1.0). However, the OAH-affected hips had 4% higher, 8% lower, and 5% lower BMD than the corresponding control hips at the FN, TR, and TH, respectively (Table 2). In addition, although not statistically significant, the OAH-affected limb's heel BMD was 2% lower. The position of the FN ROI was not affected by any osteophytes.

**Table 2.** Bone mineral density of the proximal femurs and heels, and Z-scores. Values are mean (SD)

	n	OAH limb	Control limb	Δ (%) **^a^**	p-value **^b^**
BMD-FN	37	1.05 (0.14)	1.01 (0.13)	4.1%	<0.001
BMD-TR	37	0.82 (0.14)	0.89 (0.13)	–8.3%	<0.001
BMD-TH	37	1.01 (0.14)	1.06 (0.14)	–4.6%	<0.001
BMD-Heel	38	0.54 (0.08)	0.55 (0.09)	–1.7%	0.3
Z-FN	37	0.8 (1.2)	0.4 (1.0)	n.a.	n.a.
Z-TR	37	–0.2 (1.2)	0.4 (1.2)	n.a.	n.a.
Z-TH	37	0.1 (1.1)	0.5 (1.1)	n.a.	n.a.

**^a^** Percentage difference OAH–Control

**^b^** Paired t-test

BMD in g/cm**^2^**

Z-scores: sex- and age-matched reference values adjusted for weight

n.a.: not applicable.

*Bone mineral content.* The OAH-affected hips had 18% higher, 18% lower, and 5% lower BMC than the corresponding control hips at the FN, TR, and TH (Table 3).

**Table 3.** Bone mineral content and projected bone area at the proximal femur. Values are mean (SD)

Part**^a^**	OAH limb	Control limb	Δ (%) **^b^**	p-value **^c^**
BMC-FN (g)	6.2 (1.4)	5.3 (0.9)	18%	<0.001
BMC-TR (g)	10.9 (3.4)	13.3 (3.7)	–18%	<0.001
BMC-TH (g)	36 (7.4)	37.9 (7.1)	–4.5%	<0.05
Area-FN (cm**^2^**)	5.9 (0.8)	5.2 (0.6)	13%	<0.001
Area-TR (cm**^2^**)	13.2 (2.9)	14.9 (2.8)	–11%	<0.001
Area-TH (cm**^2^**)	36 (4.3)	36 (4.0)	–0.1%	0.9

**^a^** FN: femoral neck; TR: trochanteric region; TH: total hip.

**^b^** Percentage difference OAH–Control

**^c^** Paired t-test; n = 36

*Projected bone area.* The OAH-affected hips had 13% higher and 11% lower projected bone area than the corresponding control hips at the FN and TR (Table 3). There was no significant difference in projected bone area in the TH.

We assessed the DXA images of the proximal femurs of the OAH-affected and healthy sides for differences in rotation and for the presence of osteophytes that might influence proximal femur ROIs. 19 patients had similar positions for their OAH side and healthy side, whereas 16 patients had the OAH-affected femur in a more external position. One of the patients had the OAH-affected proximal femur in a more internal rotation. If the comparison was restricted to the 19 patients with similar rotation for the 2 hips, the same statistically significant changes in BMD (–6% at the TR and +5% at the FN) could be seen as when all patients were included.

*Body composition.* Regional analyses of body composition showed statistically significantly lower BMC in the OAH-affected limbs (Table 4). Also, FM and LTM as well as total lower limb mass, TLLM, showed lower values, although the differences were not statistically significant. In order to reduce the influence of the lower limb ROI size, the percentages BMC%, FM%, and LTM% were calculated. Although not statistically significant, the BMC% (–7%) was even lower for the OAH-affected limbs.

**Table 4.** Body composition of the lower limb measured with DXA for 38 patients with unilateral osteoarthritis of the hip. Values are mean (SD)

Lower limb **^a^**	OAH limb	Control limb	Δ(%) **^b^**	p-value **^c^**
BMC **^d^**	0.57 (0.11)	0.60 (0.10)	–4.8%	<0.001
FM **^d^**	4.16 (1.68)	4.23 (1.75)	–1.7%	0.06
LTM **^d^**	7.87 (1.76)	7.96 (2.06)	–1.1%	0.7
TLLM **^d^**	12.60 (2.05)	12.79 (2.49)	–1.5%	0.5
BMC% **^e^**	4.5%	4.9%	–6.8%	0.2
FM% **^e^**	32.8%	33.3%	–1.5%	0.7
LTM% **^e^**	62.7%	61.8%	1.4%	0.5

**^a^** FM – fat mass; LTM – lean tissue mass; TLLM – total lower limb mass

**^b^** Percentage difference OAH–Control

**^c^** Paired t-test

**^d^** Expressed as 10^3^ g

**^e^** Expressed as a percentage of total lower limb mass

### Rotation group

Trochanteric BMD was statistically significantly lower, –2.4%, when the proximal femur was scanned in the recommended standard position with the foot at 10–15° of internal rotation, as compared to scanning with the foot in vertical position (i.e. external rotation of the femoral neck, simulating the common fixed position in arthritic hips). For the FN and TH, BMD was similar with respect to rotation of the hip at the time of scanning (Table 5).

**Table 5.** The effect of rotation of the proximal femur on BMD of the hip (rotation group, 21 patients, 42 hips). Values are mean (SD) in g/cm^2^

Part **^a^**	Zero	External	Δ(%) **^b^**	p-value **^c^**
FN	0.87 (0.13)	0.87 (0.13)	0.3%	0.7
TR	0.72 (0.12)	0.74 (0.12)	–2.4%	<0.001
TH	0.88 (0.13)	0.88 (0.13)	–0.5%	0.3

**^a^** FN: femoral neck; TR: trochanteric region; TH: total hip.

**^b^** Percentage difference Zero–External

**^c^** Paired t-test

## Discussion

Even though the patients with OAH had high pain scores, impaired function, impaired range of motion, and lower self-reported function, we found no differences in weight bearing and hip abduction strength between the affected and the control limbs. The principal findings were a reduced BMD at the trochanter and total hip and increased BMD at the femoral neck, but without affecting heel BMD or body composition in the OAH-affected limbs. This pattern, with a decrease at the trochanter and an increase at the femoral neck, was also seen regarding BMC and projected bone area.

### Bone mineral density

There are different opinions as to whether OAH is associated with increased hip BMD. Some argue that locally increased BMD may be part of the pathogenesis of OAH and consequently lead to increased mechanical stress in cartilage during loading and joint movement (Nevitt et al. 1995, Antoniades et al. 2000). Asymptomatic patients with early radiographic signs of OAH have been reported to have an elevated FN BMD, leading to the conclusion that not only cartilage but also adjacent bone is affected in OAH (Bruno et al. 1999). Nevitt et al. (1995) assessed pelvic radiographs and DXA results in 4,855 Caucasian women and concluded that women with moderate to severe OAH have higher BMD in the hip (FN BMD, Ward's triangle, and TR BMD), spine, and appendicular skeleton than patients with OAH of grade 0–1. Arokoski et al. (2002a) found significantly higher BMC of the femoral neck (18%) in OAH patients than in healthy controls, but there was no difference in BMD. In line with our findings, they showed increased projected area in the femoral neck, and reduced projected area and BMC of the trochanter, with higher radiographic scoring of OAH. This was explained by a larger femoral neck size as measured with MRI.

Twins with osteophytes had 4% higher FN BMD than unaffected co-twins (Antoniades et al. 2000). A similar increase in FN BMD (of 3–8%) for patients with OA of the hip and/or knee, has been reported from a large population-based study (Burger et al. 1996). In addition, statistically significant increases in FN BMC (8%) and TR BMC (13%), but not in FN BMD and TR BMD, was found in 99 women treated surgically for hip or knee OA (Sandini et al. 2005). A case-control study in which 27 men with unilateral or bilateral OAH were compared to 30 healthy controls revealed similar BMD at proximal femur ROIs and heels (Arokoski et al. 2002a). OAH is often a bilateral disease at different stages (Danielsson and Lindberg 1997). Comparison of BMD, muscle strength, and body composition to the so-called “unaffected” side is therefore not uncomplicated. In our study, only patients with unilateral OAH were included and the patients were followed for 5 years without developing OAH of the control limb that required total hip arthroplasty.

Furthermore, the issue of rotation of the proximal femur has not been addressed in previous reports but is a matter of concern (Girard et al. 1994, Goh et al. 1995). However, the increase in FN BMD and the reduction in TR BMD in our study remained stable and statistically significant when the analysis was restricted to individuals with similar rotations in DXA images. The results from the rotation group showed that a limited external rotation resulted in a 2.4% increase in TR BMD. This indicates that our reported reduction in TR BMD is more likely to have been an underestimation rather than an overestimation of the true value. Although normal Z-scores were recorded for all proximal femur ROIs, an 8% reduction in TR BMD and 1.0 lower Z-score at the TR compared to the FN might imply that the trochanteric region is the weak part of the proximal femur, and that it is prone to fractures when exposed to trauma (Fox et al. 2000).

The similar heel BMD results for the OAH-affected limbs and controls may reflect the similarity recorded in weight bearing between the limbs, as has been reported previously (Arokoski et al. 2002a).

Many factors including age, sex, BMI, BMD, medication, tendency to fall, other diseases, and infections contribute to the risk of fracture. Hip geometry is another factor that has been thoroughly investigated and shown to influence hip fracture risk (Bergot et al. 2002, Ulusoy et al. 2008). The higher BMD in OAH may prevent fractures that may, however, be counteracted by increased body sway and lower quadriceps strength and a higher risk of falls (Jones et al. 1995, Arden et al. 1996, 1999). In addition, men with OAH have been found to have reduced absolute volume of trabecular bone in the greater trochanter (Obrant 1984). Supported by these data, our findings with relatively lower BMD and Z-scores at the trochanteric region compared to the femoral neck offer an explanation as to why OAH patients—if they sustain a hip fracture—are more likely to suffer from a trochanteric type of fracture.

Although the patients in our study had severe symptoms, the affected limbs had similar hip abductor strength and weight bearing to the control limbs. This could possibly explain why no side differences were found for body composition in the leg or heel BMD. There was, however, a tendency for lower limb BMC (although not statistically significant when calculated as BMC%). This contrasts with other studies that have shown reduction in hip flexion strength and lean mass (by DXA) (Madsen et al. 1997), decrease in muscle mass and bone mass of the thigh (by CT) (Adolphson et al. 1993), and lower cross-sectional muscle area (by MRI) (Arokoski et al. 2002b).

This study had several limitations. Measurement of weight bearing was done in hospital, and not in a normal day-to-day environment. Only a few steps were registered and although the patient was unaware of when registration was done, he or she may have put more weight on the OAH leg than during normal daily walking activity. Measurement of muscle strength in patients with pain is difficult. Pain may limit muscle strength in daily life, but we were not able to demonstrate this. Our study population had a mean age of 55 years, and was considerably younger than the average hip fracture patient. The low mean age in our study was due to the fact that they were recruited for a non-cemented THA, and thus not representative for OA in general. The differences detected in BMD, BMC, and projected bone area will of course change with time, and it is difficult to speculate about circumstances at hip fracture age. However, a linear age-related decrease in BMD of similar magnitude has been found for all three ROIs in normal women between 30 and 90 years of age (Duboeuf et al. 1991).

Our study also had several strengths. We performed a bilateral study on strictly unilateral OAH cases with the healthy side as the control. This eliminates all inter-individual differences between cases and controls. Furthermore, we combined DXA of the proximal femora and heels bilaterally with regional analyses of total body measurements in the same group of patients. The possible influence of rotation on BMD was addressed.

In summary, osteoarthritis of the hip increases femoral neck BMD and reduces trochanteric and total hip BMD without affecting BMD of the peripheral skeleton, or body composition. The BMC and projected bone area is increased at the femoral neck and decreased at the trochanter. Our findings may be one explanation for the epidemiological fact that we see very few femoral neck fractures in patients with osteoarthritis of the hip.
